# A Case of Puerperal Convulsions Relieved by Blood-letting

**Published:** 1852-01

**Authors:** Benj. C. Drury

**Affiliations:** Breckinridge County, Ky.


Art. III.—*A Case of Puerperal Convulsons relieved by Blood-letting.* By Benj. C. Drury, M. D., of Breckinridge County, Ky.

Mrs. D., æt. about 30 years, who had borne three living children, and miscarried several times, being near another confinement, was seized on the morning of the 9th of November with a convulsion, after having suffered with headache and vomiting for a short time after waking. I found her with a hot skin, hard, bounding pulse, insensible and speechless. The convulsions recurred with tolerable regularity once every hour until blood was drawn by Dr. Board, after which the intervals were increased to two hours. During the paroxysms, her tongue had been badly lacerated by her teeth, and was much swollen. Her extremities and general surface presented a tumid appearance from distension of the blood vessels. It was at once decided that more blood should be extracted, and accordingly the patient was bled from the arm to the extent of half a gallon. When this quantity had been drawn, her face became blanched, her pulse grew soft, and the surface relaxed. After this, she had no return of the convulsions, though it was nine hours before entire cousciousness was restored. A dose of calomel was administered with some difficulty, which operated well. Labor came on in a few hours, and the lady was delivered of a large still-born child. On the 1st of December, two days after, I found her doing well, and she recovered without any untoward symptom.

It is due to Dr. George Thompson, of Tennessee, to say, that I was encouraged to use the lancet so freely in this case, by his practice in similar cases. My impression is decided, that our patient owes the preservation of her life to the copious blood-letting, which we should hardly have had the boldness to practice but for the experience of Dr. Thompson.

December 15, 1851.

